# Giant Lactating Adenoma With Fibroadenomated Changes

**DOI:** 10.7759/cureus.14706

**Published:** 2021-04-27

**Authors:** Sherif Monib, Kelvin Chong

**Affiliations:** 1 Breast Surgery, West Hertfordshire Hospitals NHS Trust, St Albans, GBR

**Keywords:** benign breast disease, lactating adenoma, fibroadenomatoid change, pregnancy related breast changes

## Abstract

Lactating adenomas (LAs) are uncommon benign breast tumors that typically occur in the late pregnancy or lactation period and are among the most prevalent breast lesions during puerperium. They commonly present with a painless, rapidly growing, large, mobile breast lump either late in pregnancy or the postpartum period. Despite being a condition, a core biopsy is almost always required to exclude malignancy.

We are presenting a case of a 34-year-old patient who was referred to our unit with a progressive increase in size of the pre-existing right breast lump that has been there before pregnancy. Due to the massive increase in size in a short period, the lump was removed shortly after delivery with an acceptable cosmetic outcome.

## Introduction

Benign breast diseases represent a fair amount of referrals to breast units; some of which are common during pregnancy and lactation period. A lactating adenoma (LA) is a rare benign breast tumor that tends to present during pregnancy or shortly after as palpable, well-circumscribed, lobulated masses that vary in size. Despite its benign nature, it might develop aggressively and can be misdiagnosed as breast cancer [1].

A LA is commonly seen in young primiparous women during their second or third decades of life; it is thought to be attributed to hormone-induced physiological and pathological changes [2]. In some cases, they might cause clinical concern due to their progressive course of growth. Hence triple assessment is mandatory even if the clinical impression is lactational adenoma.

Our case report is unique as LA which developed in relation to a pre-existing tiny breast lesion, progressively increased in size to reach such an enormous size. Our case was reported in line with Surgical CAse REports (SCARE) criteria [3].

## Case presentation

We are presenting a case of a 34-year-old female who was referred to our breast unit with a progressive increase in size of the right breast lump. Her past medical history and family history were irrelevant. General examination was unremarkable, and breast examination revealed a right breast 50 mm oval, firm mass at 6 o’clock location, with no other palpable suspicious lumps in both breasts and axillary or supraclavicular lymph nodes.

**Figure 1:**
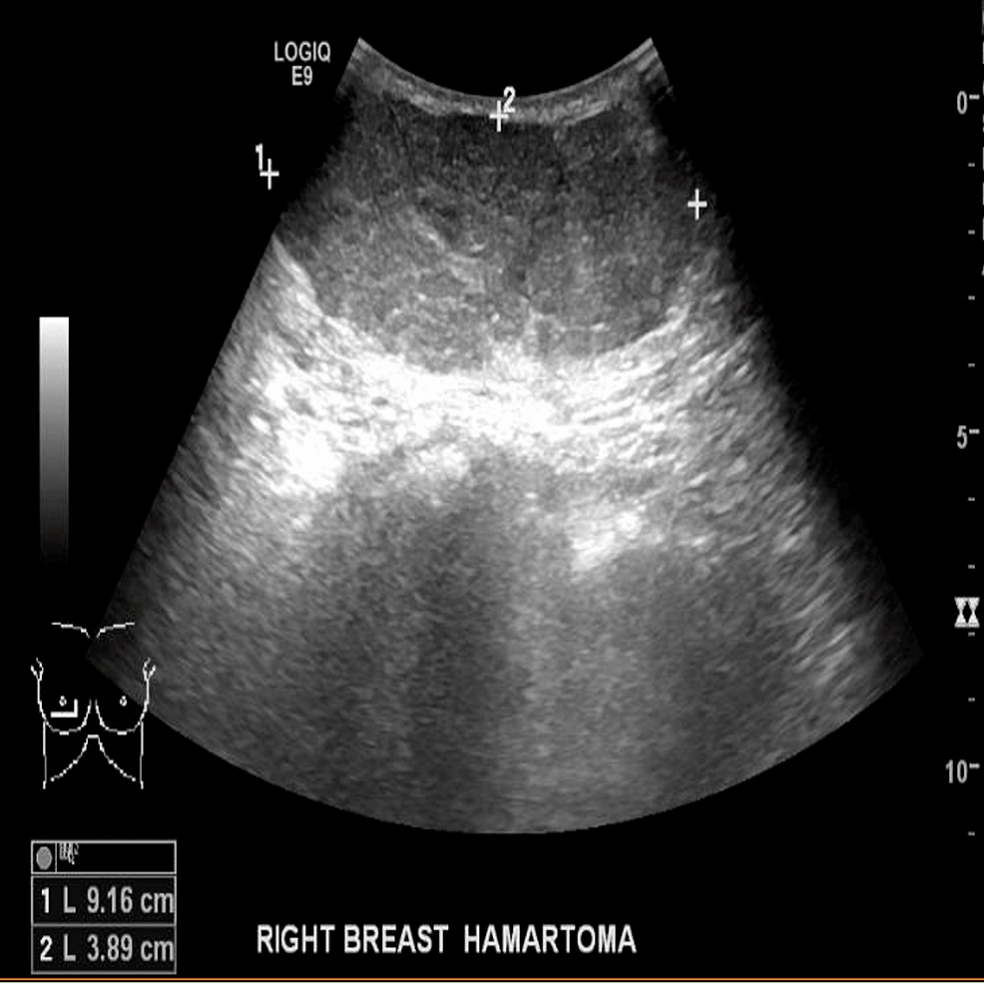
Right breast USS prior to pregnancy showing a U3 52 mm, heterogeneous hypoechoic lesion. USS, ultrasound scan

**Figure 2:**
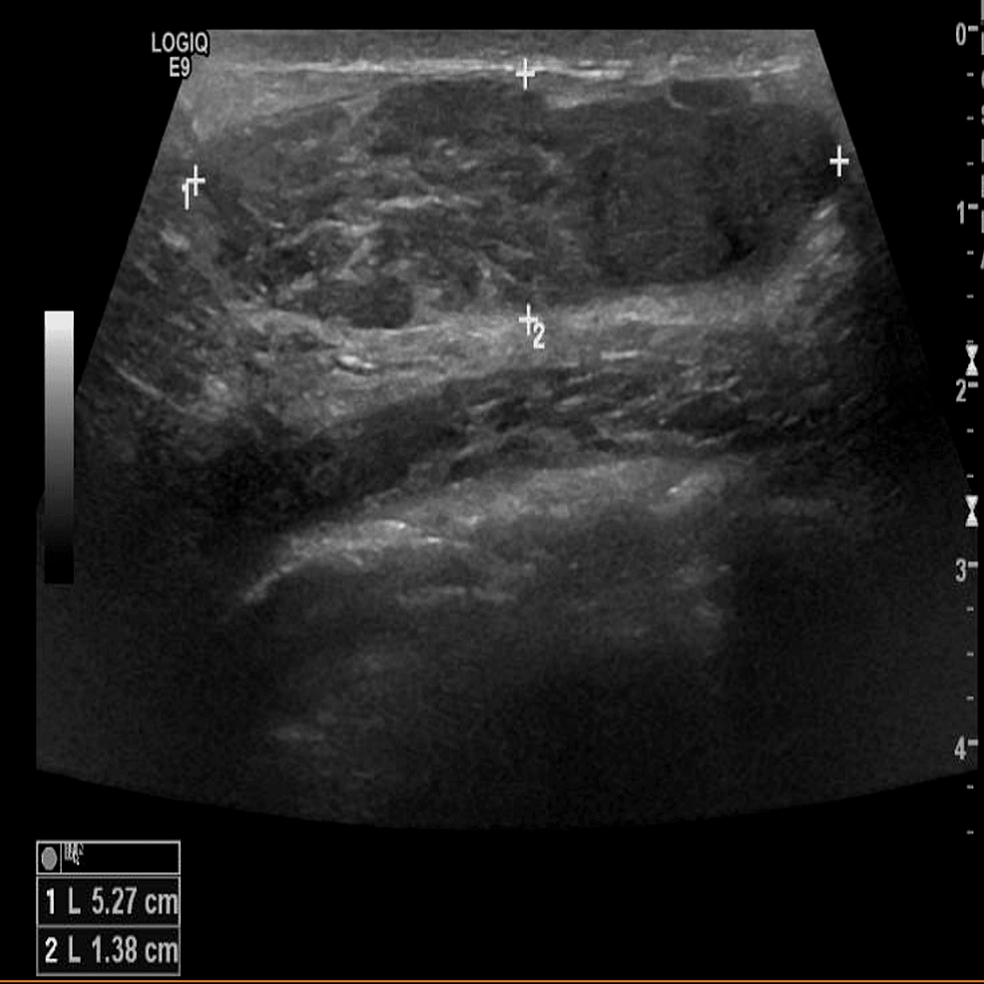
Right breast USS at 12 weeks showing a considerable increase in the size of previous lesion to 91 mm. USS, ultrasound scan

Right breast ultrasound scan (USS) showed a U3 52 mm, heterogeneous hypoechoic lesion (Figure 1), which was suggested to be a hamartoma on histopathological examination six months earlier. Our multidisciplinary team advised discussing surgical excision options or a follow-up USS in three months with the patient who opted for the latter option. She was then seen in the clinic four-month afterwards with a follow-up USS which showed a considerable increase in size to 91 mm but in keeping with the previous findings (Figure 2).

 

The patient was then 11 weeks pregnant; due to the pregnancy status, our multidisciplinary team advised for close clinical follow-up and excision after delivery.

**Figure 3:**
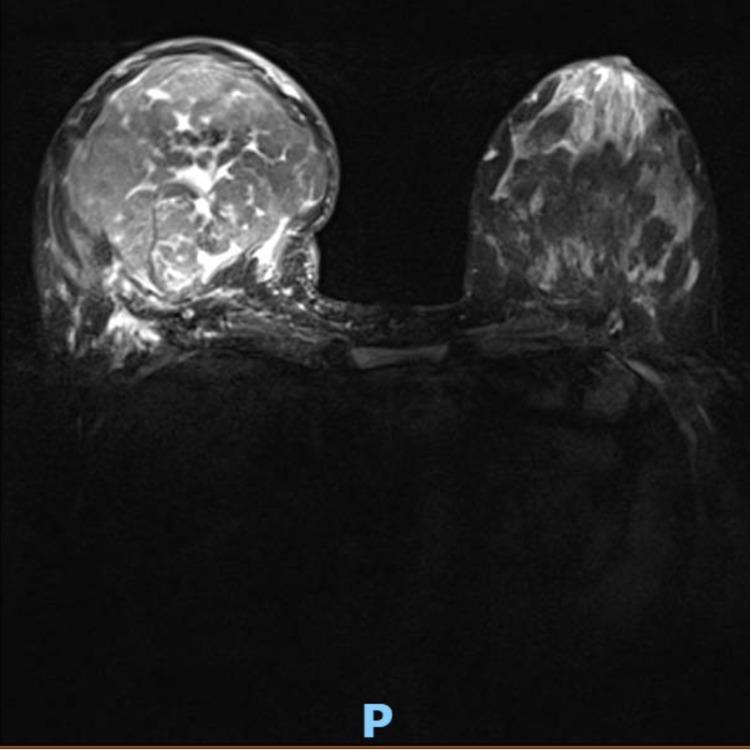
Bilateral breast MRI at 23 weeks showing 11.7 mm well-circumscribed hamartoma occupying the lower half of the breast with vivid heterogeneous enhancement and some central unenhanced dendritic-like areas.

Subsequent clinical examination at 23 weeks of pregnancy, the mass was found to continue to grow in size; at that point, a breast MRI was recommended and carried out. It showed 117 mm well-circumscribed hamartoma occupying the lower half of the breast with vivid heterogeneous enhancement and some central unenhanced dendritic like areas (Figure 3).

**Figure 4:**
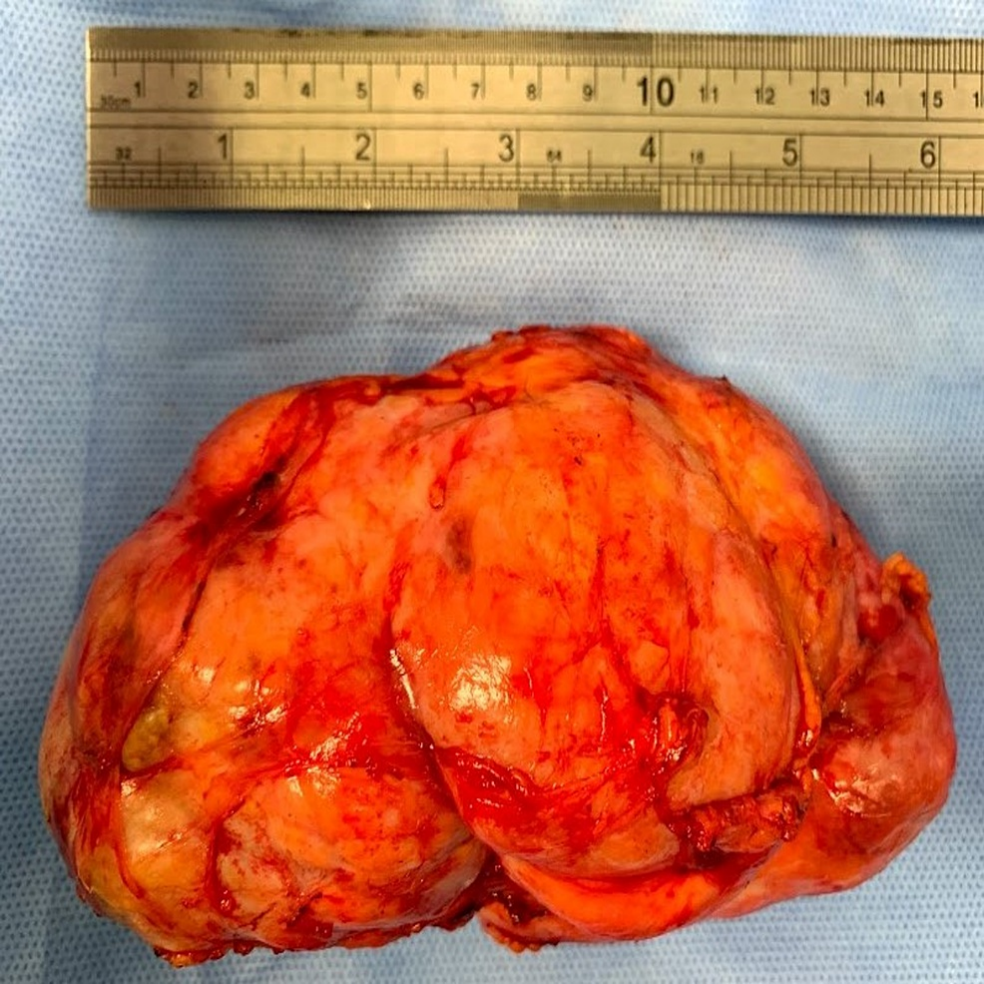
Histopathology specimen showing a 145 mm x 75 mm x 60 mm, oval, bosselated, firm lump.

Following normal vaginal delivery, the patient decided not to breastfeed, to proceed with surgery in line with our multidisciplinary team (MDT) recommendations. Preoperative breast examination revealed right breast 150 mm lower pole mass causing an obvious discrepancy in size and shape between breasts. The hamartoma was fully excised under general anesthesia via an inframammary crease revealing a 381 g, 145 mm, oval, bosselated, firm lump (Figure 4).

The patient was discharged the following day with no immediate postoperative complications. She was then seen in the clinic two weeks after, where breast examination revealed good cosmetic result, yet a slight dent seen at the lower half of the breast was noted. Final histology revealed a 145 mm x 75 mm x 60 mm lactational adenoma with benign nodular fibroadenomated changes. 

## Discussion

A LA is a benign epithelial lesion of the breast which typically occurs in late pregnancy. There has been no clear consensus on whether LA is considered a distinct condition or as a result of pregnancy-induced changes from pre-existing fibroadenoma, lobular hyperplasia, or tubular adenoma [4].

Breast lesions presenting during pregnancy and lactational period are benign in 80% of patients, yet etiology and pathogenesis are still unclear. Some authors suggested that pregnancy-related hormones can enhance the growth of pre-existing benign breast lesions as fibroadenomas [5] which was the case with our patient.

Pregnancy-related breast changes such as stromal involution, ductal dilatation, and glandular proliferation make the clinical and radiological assessment a bit challenging [6]. Due to radiation risk and increased breast density during pregnancy, breast USS is considered the primary diagnostic modality for assessing breast lesions during pregnancy and lactation. Yet, mammography and MRI might be required in selected cases [7]. This was the case with our patient as we did an MRI at 23 weeks of pregnancy to rule out a possible change of the lump's nature due to rapid progressive growth.

Parnes et al. highlighted that LA possible mammographic findings include a well-defined, lobulated mass with fat density or fluid-fat level [8]. In our case, we did not do a mammogram due to the patient's young age and pregnancy. Histologically LA appears as well-circumscribed, lobulated, solitary or multiple, with areas of necrosis; while microscopically, it is seen as well-circumscribed nodules of tightly packed lobular acini lined by cuboidal epithelial cells with markedly vacuolated cytoplasm containing lipid-rich material, hobnail appearance, and eosinophilic secretions, within the acini [9-10].

Simple excision or enucleation is always the recommended surgical option for lesions that do not resolve spontaneously [2]. In some cases, reconstructive options might be used to avoid breast deformity. Ter Louw et al. presented a case of giant juvenile fibroadenomas, which required skin envelope reduction, lower pole reconstruction, and free nipple grafting to achieve symmetry with the other breast [11].

Some surgeons have also used bromocriptine, a dopamine agonist, to reduce the tumor's size before excision [12]. In our case, we opted to proceed with the surgical option without the need to use bromocriptine as the lesion was progressively increasing in size throughout and after pregnancy.

A LA is a common entity of benign breast disease, yet etiology and management guidelines are not clear; we recommend an initial triple assessment to ascertain the initial diagnosis and surgical excision for persistent lesions to delineate the final diagnosis.

## Conclusions

A LA is a benign tumor that typically occurs during pregnancy or lactation period. Despite limitations of imaging modalities due to pregnancy and pregnancy-related breast changes triple assessment, including core biopsy, stays the mainstay for diagnosis.

Close follow up using different imaging modalities, if needed, is crucial to pick up any change of nature. Surgical excision after pregnancy might be required to confirm the diagnosis of persistent lesions.

Further studies, including large sample size, will help develop robust data to stratify this group of patients' management guidelines to reach the best outcome.

## References

[1] Phung HT, Nguyen LT, Nguyen HV, Nguyen CV, Nguyen HT (2020). Aggressive lactating adenoma mimicking breast carcinoma: a case report. Int J Surg Case Rep.

[2] Ravikanth R, Kamalasekar K (2019). Imaging of lactating adenoma: differential diagnosis of solid mass lesion in a lactating woman. J Med Ultrasound.

[3] Agha RA, Borrelli MR, Farwana R, Koshy K, Fowler AJ, Orgill DP (2018). The SCARE 2018 statement: updating consensus Surgical CAse REport (SCARE) guidelines. Int J Surg.

[4] Barco Nebreda I, Vidal MC, Fraile M, Canales L, González C, Giménez N, García-Fernández A (2016). Lactating adenoma of the breast. J Hum Lact.

[5] Vashi R, Hooley R, Butler R, Geisel J, Philpotts L (2013). Breast imaging of the pregnant and lactating patient: physiologic changes and common benign entities. Am J Roentgenol.

[6] Maranhão N, Maranhão B (2021). Practical considerations for evaluation of images of the breast during pregnancy and lactation. Radiol Bras.

[7] Deepak KS, Bharat MP, LD NK (2020). Evaluation of breast lesion by ultrasonography during pregnancy and lactating women. Int J Health Clin Res.

[8] Parnes AN, Akalin A, Quinlan RM, Vijayaraghavan GR (2013). AIRP best cases in radiologic-pathologic correlation: lactating adenoma. Radiographics.

[9] Samaneh A. Motanagh, Kristen E (2021). PathologyOutlines.com. Muller. Pathology Outlines.com.[accessed.

[10] Dabbs DJ (2016). Breast Pathology E-Book.

[11] Ter Louw RP, Bruce SB, Nahabedian MY (2017). Partial breast reconstruction with Goldilocks technique after excision of giant fibroadenoma: a case report. Plast Reconstr Surg Glob Open.

[12] Taib NA, Rahmat K (2020). Benign disorders of the breast in pregnancy and lactation. Adv Exp Med Biol.

